# Spinal Anesthesia Management in a 30-Year-Old Patient with Progeria Syndrome: A Case Report

**DOI:** 10.5812/aapm-147344

**Published:** 2024-06-19

**Authors:** Maryam Vosoughian, Faramarz Mosaffa, Shide Dabir, Mastaneh Dahi Taleghani

**Affiliations:** 1 Department of Anesthesiology & Critical Care, Taleghani Hospital, Anesthesiology Research Center, Shahid Beheshti University of Medical Sciences, Tehran, Iran; 2 Department of Anesthesiology & Critical Care, Anesthesiology Research Center, Akhtar Hospital, Shahid Beheshti University of Medical Sciences, Tehran, Iran

**Keywords:** General Anesthesia, Progeria Syndrome, Spinal Anesthesia

## Abstract

Progeria syndrome is a rare genetic disorder resulting in premature aging. General anesthesia is very challenging in these patients due to difficult intubation and age-related comorbidities. We describe spinal anesthesia management in a 30-year-old man with progeria syndrome. To our knowledge, this is the first report on using spinal anesthesia in this group of patients.

## 1. Introduction

Progeria syndrome is a rare genetic disorder caused by a mutation in the LMNA gene, which encodes lamin A/C proteins that play a crucial role in maintaining the integrity of the cell nucleus, leading to premature aging (1). Patients with this condition present challenges in anesthetic management, including difficulties with intubation (2) and a high risk of cardiovascular (3) and cerebrovascular diseases (4). Preoperative assessment, including airway examination and meticulous preparation for potential cardiovascular events, is essential in patients with progeria syndrome (1). The limited cases reported on anesthesia management in these patients have primarily involved general anesthesia. This is the first report on the use of spinal anesthesia in a progeria patient.

## 2. Case Presentation

**Figure 1.**
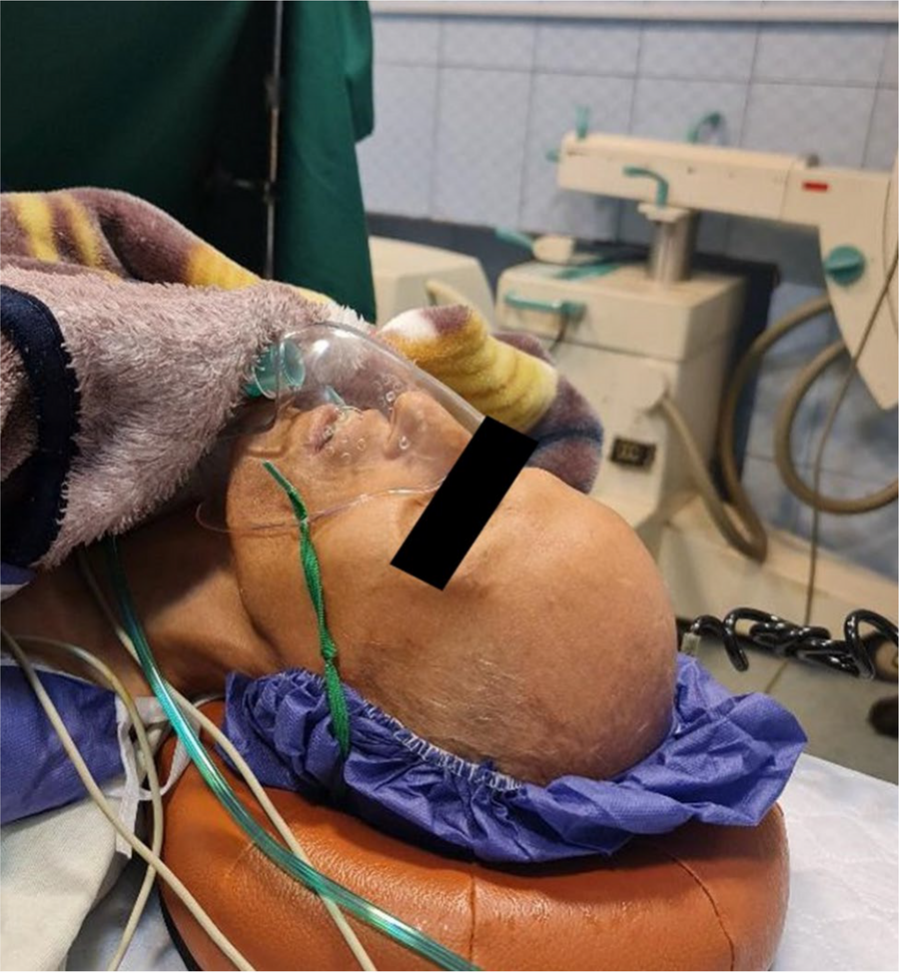
Patients’ feature

We describe a 30-year-old man with progeria syndrome who presented with severe pain and swelling in his left knee joint following trauma, ultimately diagnosed as septic arthritis requiring surgical intervention. The patient had a height of 151 cm and a weight of 20 kg. He exhibited characteristic features of progeria, such as decreased subcutaneous tissue, alopecia, restricted mouth opening, decreased neck flexibility, hearing impairment, and hoarseness (Figure 1). He had a history of hypertension and was treated with losartan 25 mg daily.

**Figure 2.**
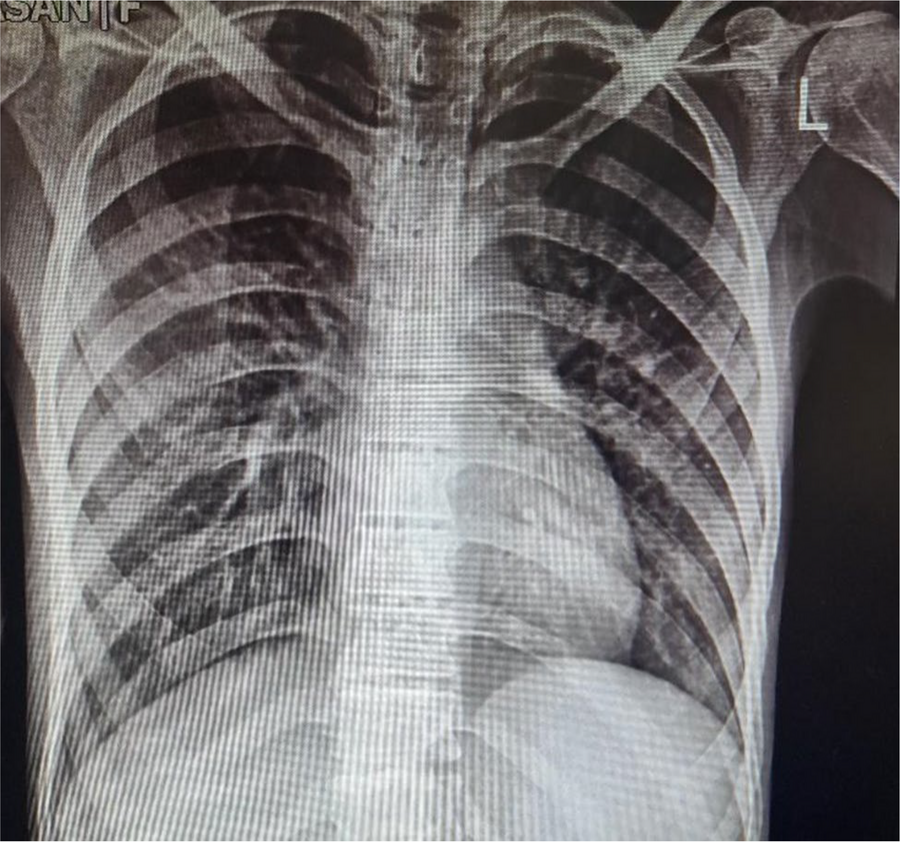
Patients’ chest X-ray

Echocardiography showed a 55% left ventricular ejection fraction. Blood test results, electrocardiogram, and chest X-ray (Figure 2) were unremarkable.

The patient underwent left knee pus drainage surgery. Standard monitoring (electrocardiogram, noninvasive blood pressure, and pulse oximetry) was applied, and a peripheral intravenous line was established. Due to airway issues, ultrasound-guided combined sciatic and femoral nerve blocks were performed using 7 mL of 1.5% lidocaine (Saman Pharmaceutical Company, Mashhad, Iran) with epinephrine for each block through the anterior approach. The patient's surgery was completed without any complications.

Two days later, joint irrigation and debridement were performed under spinal anesthesia. Before anesthesia, his blood pressure was 160/95 mmHg, pulse rate 110/min, and room air O_2_ saturation 95%. Spinal anesthesia was administered using 7.5 mg of bupivacaine 0.5% (1.5 mL) (Varrian Pharmed, Tehran, Iran) with a Quincke 25-gauge spinal needle (Dr. Japan Co. Ltd, Tokyo, Japan) at the L3-L4 level. Multiple attempts were needed due to age-related skeletal anatomy. The drug amount was adjusted based on the operation's duration. The sensory level reached T2, leading to hypotension (blood pressure decreased to 95/63 mmHg) and a heart rate of 115/min, which was safely treated with fluid resuscitation and ephedrine administration. The patient also experienced a cold sensation and shivering, which were managed by a forced air warmer. The patient was discharged from the operating room in good general condition and with stable hemodynamics.

The next three surgeries for joint drainage and debridement were performed under spinal anesthesia at three-day intervals using 5 mg of bupivacaine 0.5% (1 mL) without any complications. Each time, sensory block levels reached T10, maintaining stable hemodynamics throughout.

## 3. Discussion

We described spinal anesthesia management in a 30-year-old man with progeria syndrome. To our knowledge, this is the first report on using spinal anesthesia in this group of patients. Anesthesia management is challenging for anesthesiologists in progeria patients, particularly regarding airway management. Therefore, we chose regional anesthesia to minimize the adverse effects of general anesthesia. We performed sciatic and femoral nerve blocks in the first operation and spinal anesthesia in the subsequent surgical procedures.

However, the use of spinal anesthesia in progeria syndrome requires careful consideration, including local anesthetic dose adjustments and close monitoring. In our patient, the initial dose of 7.5 mg of bupivacaine 0.5% for spinal anesthesia resulted in an increased block height and significant hypotension. These adverse effects were resolved by reducing the dose of bupivacaine to 5 mg in subsequent surgical procedures. Additionally, age-related changes in the spine of progeria patients can make performing spinal anesthesia technically difficult. We encountered difficulties in performing spinal anesthesia each time. Furthermore, the lack of subcutaneous tissue predisposes these patients to hypothermia, necessitating precautions to prevent this complication during procedures.

Individuals with progeria syndrome typically have a short life span, with an average life expectancy of 14 years. Our patient was a rare case who lived much longer. Increased vigilance and care may potentially extend life expectancy in such patients. Additionally, a limited number of reports on anesthesia management in progeria cases exist, all of which were performed under general anesthesia. Various approaches for laryngoscopy, intubation, and sedation have been reported in these cases.

Nguyen and Mayhew (5) performed a 'blind' oral intubation guided by respiratory sounds. Liessman (6) described dental surgery conducted with laryngoscopic-assisted fiberoptic intubation using a guide wire and bougie. Russo-Menna and Arancibias (7) detailed an uneventful traditional laryngoscopy. Hansda et al. (8) successfully intubated a patient on the third try using direct laryngoscopy and an intubating stylet. Espandar et al. (9) effectively reduced shoulder and hip dislocations in a sedated patient with spontaneous breathing. Similarly, Alevizou et al. (10) described a fiberoptic orotracheal intubation with sedation and spontaneous breathing. Vreeswijk et al. (11) induced anesthesia by maintaining normal breathing with sevoflurane or propofol and fentanyl. Oliveira et al. (4) administered an infusion of dexmedetomidine and ketamine while preserving spontaneous ventilation for laryngoscopic evaluation and then used propofol and succinylcholine for intubation. They also performed ultrasound-guided femoral nerve and lateral femoral cutaneous nerve blocks for postoperative pain control.

### 3.1. Conclusions

Based on our experience, spinal anesthesia is safe in progeria patients, provided that caution is taken in choosing the appropriate dose of local anesthetic and patients are carefully monitored. Special attention should also be paid to age-related comorbidities.

## Data Availability

The dataset presented in the study is available on request from the corresponding author during submission or after publication.
